# The Possibilities of the Tooth-Brush

**Published:** 1903-06

**Authors:** B. F. Arrington

**Affiliations:** Goldsboro, N. C.


### THE POSSIBILITIES OF THE TOOTH-BRUSH.[1]

BY DR. B. F. ARRINGTON, GOLDSBORO, N. C.

As a health-preserving factor (preservative and restorative) there possibly is no one agent, implement, or medicinal remedy in the catalogue of remedies recorded in materia medica, nor any one or one hundred of the numerous patent and proprietary remedies with which the country is at present so mercilessly supplied, that can be used to such beneficial advantage for prevention of disease and for the preservation of teeth and gums as the tooth-brush, if carefully and systematically applied several times daily from early childhood to mature and advanced age. A simple, easy practice, requiring not more than three or four minutes daily, and not half

---

[1] Read at the annual meeting of the Alumni Clinic, Nashville, Tenn.

so troublesome or taxing as the washing of face and hands, combing hair, or caring for finger-nails.

Had such practice been introduced and generally indulged, commencing as far back as seventy-five years ago, there would have been no necessity for the establishment of a dental college in this country, and in all probabilty there would have been none, whereas to-day we have fifty or more and others in prospect. A greater number than was ever dreamed of by Hayden and Harris, the original instigators and organizers of the Baltimore Dental College, the first and oldest in existence, and which dates back less than sixty-five years. If the views and arguments of those level-headed, practical men had been respected and their earnest pleadings for the establishing of a chair of Oral Surgery in medical colleges been heeded, what a blessing in all probabilty would have come to the human family! Mouths comparatively free from taint, healthy gums, and well-preserved teeth would to-day be the result and rule and not the exception. As it is, this, the second day of February, 1903, with many dental colleges, a National Dental Association, with innumerable Dental Societies in States and cities discussing ways and means for best results from practice, there is not one mouth in fifty to be found that can be pronounced healthy; teeth decay and are extracted by thousands when one should not be; and disease of teeth and gums prevail and continue unabated with rare exception, as before dental colleges were established,—a regretable and deplorable state of things. What is the cause and what is the remedy? Filthy mouths through neglect of proper instruction and free use of tooth-brush is unquestionably the chief cause, and the free use of the brush with plenty of water is the preventive remedy for relief. There are other causes for diseased conditions of teeth and gums, and for the loss of teeth, but of much less magnitude. The neglect of use of tooth-brush is the chief cause of unhealthy mouths, foul breath, and loss of teeth, and for much of the conspicuous, objectionable display of gold in mouths at this advanced period of dental practice, such as display advertising fillings and gold crowns on front teeth, a popular and much indulged practice at present and for some years past, and has brought reproach and shame upon the profession, with hurtful results. Less gold and more tooth-brush would be better for the profession and for the public.

Our country, from Maine to Mexico and from ocean to ocean, is flooded with germ-destroying (so termed) mouth-washes, creams, soaps, etc., to kill bacteria, to preserve teeth from decay, and to keep the gums in a healthy condition, and so far not one is definitely known to be reliable and will accomplish when used the results proclaimed. No remedy yet offered is of sufficient strength to kill a microbe and prove harmless to the soft tissues of the mouth, yet the money goes for the highly endorsed and lauded remedies, and they are most extravagantly and unreasonably used, when a judicious use of the tooth-brush and water in quantity would more effectively dislodge and expel from the mouth any hurtful excess of germ products and do no injury whatever to the soft tissues and nerve tone.

If the one-hundredth part of the time, talent, and money invested in the production and introduction of useless mouth-washes and other preparations designed for the destruction and check of germ detriment in the mouth had been given to improvement in the patterns and make of tooth-brushes, suited to the wants of all classes in a community, with practical instruction when and how to use them, a thousand-fold more good would have been accomplished in the interest of healthy mouths and strengthened constitutions, with infinitely less labor and fatigue to dentists, and at less cost with better results to patrons.

Many persons who use freely of the numerous remedies on the market for mouth treatment preserve and keep their gums and teeth in a favorable condition, but the same result with these persons could be as effectually accomplished by the simple application of tooth-brush and water as above suggested. To be convinced is to make the experiment and watch results. The results will be satisfactory, and the use of the tooth-brush and water without medicated mouth-washes and other preparations will grow in favor daily. It is the tooth-brush that does the good, removes hurtful impactions of unwholesome environments from the teeth, keeps the gums in a healthy condition, the teeth exempt from excess of caries, and the tongue freed of germ-breeding accumulations and bad odors, and makes easy for expulsion by force of water all objectional débris with dislodged bacteria.

If there is an antitoxin that will weaken and prevent germ influence for evil in the mouth without risk of injury to the soft tissues, it is the tooth-brush.

According to papers published in dental journals, based upon the theories and teachings of scientists and distinguished dentists and physicians, not only in this country but abroad, the mouth is the natural incubator for most all germ products that do injury to the human system, and it is a non-disputed fact that no germ-destroying remedy for mouth use of sufficient strength to counteract germ effect for evil in the mouth without injury to the soft tissues has yet been produced; therefore the majority of the many remedies offered are worthless and should be discarded, and for the result desired rely upon the tooth-brush and water.

To check the ravages of disease in the mouth, caries of teeth, diseased gums, peridental membrane, waste of alveolar process, loosening and loss of teeth, the effect of local causes, mostly unhealthy environments, we must resort to and rely chiefly upon the frequent, systematic use of the tooth-brush. It is, of all remedies, the most effectual and trustworthy.

Tooth-brushes of practical pattern and varied quality, with prices regulated to meet the wants of all classes, poor and rich, young and old alike, is now more greatly needed than continuation and increase of drugs for mouth use.

As practical evidence of the beneficial influence as a disease preventive and health-preserving factor the tooth-brush has, when systematically used, mention of the fact is made that in cities and towns where dentists prevail and have supervision and care of children's teeth from early childhood to maturer years, and instruct in the use of the tooth-brush, the frequency of caries in teeth and diseased gums is greatly diminished, and never advance with destructive tendency as with children of the same age in rural districts where there are no local dentists and children's teeth are neglected; and so it is in relation to Rigg's disease (pyorrhœa alveolaris) so called. In cites and towns, also in the country with persons who make daily use of the tooth-brush and are systematic in the care of their teeth and gums, the disease is but seldom seen, and never in a far-advanced typical state.

Another fact sustaining the advocacy for frequent use of the tooth-brush is that after treatment and cure of the disease above named, if the tooth-brush and plenty of water is used several times daily there will be no return of the disease. Experience in practice and observation of results long years after treatment justifies the assertion.

A proper use of the tooth-brush will, commencing with early childhood, preserve and hold intact the temporary teeth, free from rapid and destructive decay and necessity for extraction, and in youth and adult years excessive decay requiring large fillings and crowning would but seldom if ever occur, and plates and bridges would be so little needed that the execution of such work would pass from the hands of the general practitioner into the more skilful hands of prosthetic specialists.

With increased and intelligent use of the tooth-brush, not only would the teeth and gums be preserved in a comparatively normal state, with breath preserved free from taint, but the general health would doubtless be better preserved.

It is now conceded, I believe, that it is only the excess of germ products nourished in the mouth that does harm, destroys teeth, produces unhealthy gums, peridental membrane, and causes waste of alveolar process, loosening and loss of teeth, and in various ways weakens the constitution and shortens life. With the acceptance of such theory with facts recorded, the question very naturally arises, How are the hurtful and destructive effects to be most successfully battled with, and the harmful results checked and counteracted? Considering the failure in results from past treatment with varied mouth-washes for germ destruction, would it not be reasonable and advisable to test thoroughly the true merits and worth of the tooth-brush? Dentists must take the subject under consideration and decide for good or evil. We must weigh the subject carefully from a reasonable, practical stand-point, and make choice for the better prevention and check of disease, and for the healthful preservation of teeth and gums and the general good of the human system.

The tooth-brush is unquestionably our best and most effective prophylactic agent, and prophylactic treatment for preservation of teeth and gums is the all-important subject (in all sections) before the profession to-day, and should be the absorbing thought with all dentists until all are agreed that change in practice is requisite for better results. I have given much thought to the subject, and am free to say, in my humble judgment, that the tooth-brush is the essential prerequisite for success and universal good results in effort for preservation of teeth and gums in a condition as near normal as possible.

The following extracts from various journals are convincing and proof evident of the importance and necessity for more general and frequent use of the tooth-brush as a prophylactic agent in dental practice.

In a paper by Dr. W. H. Dalamoret, published in the *Medical Record,* it is shown how injurious an effect upon the health of the child might be produced by the septic condition of the mouth induced by caries of a deciduous set of teeth. Among other conditions he mentions " ulcerative stomatitis, necrosis of bone, enlarged lymphatic glands, which later might become the seat of tuberculous growth, by which the whole system might be infected." He also traced much gastric disturbance to the same cause. Then he insisted on the important part played by the first teeth in regard to the growth of the child, and he concluded that " the mastication of food could not be interfered with by the decay and extraction of deciduous teeth without impairing the health of the child."

In a paper entitled " Unclean Dentists," published in the *Medical Mirror,* we find it stated, " That there are more than one hundred individual germs of bacteria in the average mouth even in health is suggestive. But when we realize that the people who call upon dentists usually have diseased mouths, the possibilities of infection are almost appalling. Physicians should make the public familiar with the fact that the mouth is the port of entry for most infectious diseases, including diphtheria, scarlet fever, and consumption, and other even more dangerous specific diseases have been disseminated and carried from one to another through the medium of dental manipulations."

The dangers from unclean tongues and mouths and mouths containing decayed teeth are graphically set forth by Dr. Miller. He says, " From a neglected mouth, such as repeatedly comes under the observation of dentists, enormous quantities of bacteria must reach the intestinal tract." He has also said, " Out of the seven nutrient media for bacteria in the oral cavity, five are always present on a coated tongue." In the *Dental Cosmos,* January, 1903, he says, " It is a well-known fact, apart from the many local diseases of the mouth and teeth and their various sequences, the oral cavity serves as the port of entry for bacteria of a long list of the most fatal diseases to which the body is subject. I need only refer to tuberculosis, pneumonia, cholera, typhus, etc."

The consensus of opinion of many investigators seems to be that the coatings of the tongue are local and that the mass contains large quantities of bacteria.

Another writer has said, " With decayed teeth and diseased gums to breed bacteria in great numbers, the dorsum of the tongue still presents the largest surface for their growth, and if not cleaned once or twice daily it is one of the greatest of all places in the mouth for breeding bacteria."

In a very conservative and interesting paper, Dr. J. D. Patterson says, "It may be stated at the outset that the large majority of the most destructive dental diseases at their inception result from an unsanitary condition of the oral cavity."

Dr. J. R. Lessonon, in the *New York Medical Journal,* says, "Hundreds and thousands of people are going about with rotten teeth, carrying with them so many cesspools in their mouths, filled with fetid abominations of stinking food débris with its teeming population of micro-organisms, and the resulting toxins as concomitants, and daily swallowing these putrefactions and absorbing the pus. Many cases of septic disease are due to dental caries and to that alone. Teeth should be cleaned twice a day and always the last thing at night. A tooth-brush should be considered as a conglomeration of toothpicks, and used accordingly."

With such an array of facts and evidence of the unhealthy neglected condition of mouths, with evil results following, the necessity for greater care and cleanliness of the teeth and tongue for the prevention of disease and for the preservation of health, it must be plain to the feeblest minds for perception that the daily, frequent use of the tooth-brush is the important and essential factor for prevention in dental practice.

Dr. D. D. Smith is working on the right line (slightly extreme), and great good must come of his prophylaxis theory and teaching. He has entered the wedge that must split asunder and help to check the false practice of to-day, and cause reform in practice that will be accepted, and will be lauded as a grand feature in practice by the profession and the public. The ball is in motion, and impetus must be given it. Let all lend a helping hand and encourage the new feature in practice that must and will grow in favor daily. Work on right lines, if persevered in, will never prove a failure.

The pivotal point in dental practice has been reached; wonderful features in progress and results have been accomplished, far in advance of the most sanguine expectations and anticipations of dentists who were practising forty years ago.

The highest order of excellence attainable in manipulative skill in the design and execution of prosthetic work and in operations on the natural teeth has been accomplished. The thing now important to be done as the crowning feature of scientific skill in dental practice is to retrace steps, go back to the beginning, and commence practically on a conservative line of procedure with the tooth-brush and other suitable prophylactic means, and see what we as a profession can do to prevent and check ravages of decay of teeth, disease of gums, peridental membrane, waste of alveolar process, and loss of teeth, with a view thereby to do away with necessity for excessive treatment of gums and extreme operations of patch-up and restorative work in the mouth that is now so generally daily indulged, with no abatement of caries or disease of soft tissues.

We should, as members of the dental profession, write, talk, and reason together, with a laudable single object in view,—the best line of practical, conservative preventive practice for the general good and benefit of our patrons and the public, and for professional advancement, harmonize and work to secure healthy mouths, well-preserved teeth and gums, and pure breath,—unquestionably a possibility if right instruction is given and the tooth-brush is more freely used. The experiment should be made, and there is no better time to begin than now. Let us, if possible, execute professional work on higher, nobler, more reasonable and correct lines of practice until the practice of dentistry in its broadest acceptation shall be strictly conservative and scientific, and prove in the future truly a blessing simplified compared to present practice. To make the effort is our duty, and, if faithfully made, much if not all that is desired can be accomplished by increased daily use of the tooth-brush, which must be regarded as one of our chief factors for good results in an effort for preservation of teeth, and for prevention of disease.

